# Immunocytological composition of cell walls in *Sapium glandulosum* (Euphorbiaceae) galls reveals steps in their establishment and development

**DOI:** 10.1007/s00709-026-02162-5

**Published:** 2026-02-10

**Authors:** Vinícius Coelho Kuster, Maraíza Sousa Silva, Lorena Moreira Pires Rosa, Lana Laene Lima Dias, Maísa Barbosa Santos, Denis Coelho de Oliveira

**Affiliations:** 1 Departamento de Ciências Exatas e Biológicas, Universidade Federal de São João del-Rei (UFSJ), Campus Sete Lagoas, Sete Lagoas, Minas Gerais CEP 35701-970 Brazil; 2 Instituto de Biociências, Universidade Federal de Jataí (UFJ), Campus Cidade Universitária, Jataí, Goiás, CEP 75801-615 Brazil; 3 Universidade Federal de Viçosa (UFV), Cidade Universitária, Viçosa, CEP 36570-900 Minas Gerais Brazil; 4 Departamento de Biologia, Universidade Federal de Lavras (UFLA), Lavras, CEP 37200-000 Minas Gerais Brazil; 5 Universidade Federal de Uberlândia (UFU), Instituto de Biologia, Campus Umuarama, Uberlândia, CEP 38402-020 Minas Gerais Brazil

**Keywords:** Feeding activity, Hemicellulose, Host defense, Immunocytochemistry, Pectin

## Abstract

Galls alter the tissue organization of host plants, including modifications in cell wall composition. This study investigated tissue development and cell wall dynamics in galls of *Sapium glandulosum* to identify key steps involved in their establishment. Samples of young, mature, and senescent galls, as well as nongalled leaves, were analyzed using structural and immunocytochemical approaches. For histology, samples were fixed, embedded in resin, sectioned, stained with toluidine blue, and mounted with Entellan^®^. For immunocytochemistry, resin-embedded samples were tested for epitopes of cell wall proteins, pectins, and hemicelluloses using antibodies. The leaves of *S. glandulosum* are glabrous, hypostomatic, and exhibit dorsiventral mesophyll. Gall development alters the typical leaf morphogenetic pattern, giving rise to structures with a parenchymatic cortex. In young galls, hypertrophy and hyperplasia were observed, followed by tissue maturation in mature galls. Senescent galls showed signs of cytoplasmic degradation in most cortical cells. Structural modifications in the side chains of rhamnogalacturonan I and increased cross-linking of pectic polymers affect cell wall properties, playing roles in both development and defense responses. The low immunolabeling with JIM5 in young and mature galls suggests the suppressed activity of pectin methylesterases, which may reflect a strategy by which gall-inducing organisms inhibit host defense signaling. Xyloglucan epitopes were detected in the vascular bundles of mature galls, suggesting the reinforcement of cell walls and possibly supporting the feeding activity of the gall inducer. The combination of anatomical and immunocytochemical data provided a basis for understanding how gall induction modulates cell differentiation and cell wall composition in *S. glandulosum*.

## Introduction

The plant cell wall comprises a dynamic array of components, including cellulose, hemicelluloses, and pectins, as well as small amounts of proteins that perform various functions in plants (Cosgrove 2005; Lorenzo et al. 2019), such as serving as the first line of defense against invading microbes (Bacete et al. 2018). The physical properties of the cell walls depend on the chemical interactions among these components, which, together with turgor pressure, maintain structural equilibrium in plant cells (Anderson and Kieber 2020). However, biotic stress resulting from interactions between plant cells and other organisms can disrupt the cell wall and trigger multiple plant responses (Riseh et al. 2024). Galls are novel plant structures induced by the action of foreign organisms (Shorthouse et al. 2005; Harris and Pitzschke 2020) and are typically initiated by an increase in localized oxidative stress (Isaias et al. 2015). This oxidative stress is regulated by the inducing organism, which manipulates host plant tissues for its own benefit (Oliveira et al. 2016).

Gall formation involves changes to the plant’s existing morphogenetic patterns, resulting in a new cellular structure characterized by convergent processes like cell hypertrophy, tissue hyperplasia, and cellular redifferentiation (Guedes et al. 2018; Ferreira et al. 2019). These changes, which include the emergence of new cell types and modifications in cell wall dynamics, are essential for gall development and reflect the specific influence of each inducing organism. As a result, interactions between gall-inducers and hosts create significant morphological diversity, with most developmental studies traditionally focusing on anatomical and morphological aspects, and more recently on molecular mechanisms. For example, transcriptomic analyses of leaf galls across different plant–insect systems have shown shared molecular signatures, including the up-regulation of developmental genes (Takeda et al. 2019). Comparative analyses have also identified 38 common genes that are mainly involved in peptide biosynthesis, suggesting conserved mechanisms behind gall development (Takeda et al. 2019). Recent research on psyllid-induced galls has further clarified these processes through combined transcriptomic and proteomic analyses of salivary proteins (Hu et al. 2025). In *Trioza camphorae* (Hemiptera: Psylloidea), 168 potential salivary proteins were identified, including 66 conserved and 68 species-specific proteins (Hu et al. 2025). Heterologous assays in *Nicotiana benthamiana* (Solanaceae) demonstrated that three proteins influence plant physiology, indicating roles in host cell reprogramming (Hu et al. 2025). Despite these advances, the mechanisms driving gall development are still poorly understood, especially in psyllid-induced galls. This highlights the need for studies on cellular remodeling, from the cell wall to the protoplast, supported by immunocytochemical approaches.

Cellulose microfibrils are synthesized by the cellulose synthase complex in the plasma membrane, which uses UDP-glucose as a substrate (Albersheim et al. 2010). The spatial arrangement of these microfibrils in the primary cell wall determines the direction of cell elongation and hypertrophy (Kimura et al. 1999; Albersheim et al. 2010). During gall development, the reorganization of cellulose microfibrils has been associated with changes in cell shape and the formation of gall-specific structures (Magalhães et al. 2014). Arabinogalactan proteins (AGPs) are located within the cell walls and play key roles in cell signaling and adhesion during plant morphogenesis (Fincher et al. 1983; Majewska-Sawka and Nothnagel 2000). Extensins and other glycoproteins are incorporated into the insoluble microfibrillar matrix of primary cell walls, where they contribute to structural reinforcement, particularly in mature organs (Carpita and Gibeaut 1993; Brownleader et al. 1999; Albersheim et al. 2010). In galls, extensin labeling is typically associated with growth cessation, as expected (Formiga et al. 2013; Teixeira et al. 2017). However, some studies have reported unexpected extensin labeling in young galls, suggesting a more complex role during early gall development (Carneiro et al. 2014). Conversely, in *Espinosa nothofagi* (Hymenoptera) galls, extensin synthesis is inhibited, an effect that may be linked to the suppression of the host plant’s innate immune response, thereby facilitating gall initiation and development. This inhibition may also compromise cell wall reinforcement, despite the presence of tissues associated with mechanical support in these galls (Guedes et al. 2025). Another important component of cell walls, pectins, play crucial roles in regulating extensibility and growth (Showalter 1993; Mohnen 2008; Albersheim et al. 2011). Additionally, pectin functions as a signaling molecule, initiating immune responses such as antimicrobial compound production, cell wall reinforcement, and activation of defense-related genes (Saberi Riseh et al. 2024). Three main primary types of galacturonic acid-containing pectins are found in the cell wall: homogalacturonans (HGs), rhamnogalacturonans (RGs), and xylogalacturonans (XGAs) (Clausen et al. 2004; Caffall and Mohnen 2009). HGs are the most abundant, and their degree of methyl esterification significantly influences their functional role in the cell wall (Vincken et al. 2003). The degree of pectin methylesterification can also serve as an indirect indicator of the tissue developmental stage (Dolan et al. 1997). This parameter is modulated by the activity of pectin methylesterases (PMEs) localized in the cell walls, which alter wall stiffness and porosity, thereby introducing new functional properties during development (Jolie et al. 2010; Albersheim et al. 2011). The action of PMEs on HGs has been implicated in the formation of *Manihot esculenta* (Euphorbiaceae) galls, where a demethylesterification process was observed during the transition from leaf tissue to gall structures (Souza et al. 2024). A decrease in pectin synthesis signals the transition from primary to secondary cell wall formation, which is typically marked by the onset of lignin biosynthesis and the arrest of cell expansion. Lignification represents the final stage of differentiation in certain plant cells, contributing to increased wall rigidity and structural stability (Lawoko 2005). Hemicelluloses, such as heteroxylans, heteromannans, and xyloglucans, interact with cellulose microfibrils, forming crosslinks that help regulate cell wall extensibility and, consequently, cell expansion (Cosgrove 2016; Chen et al. 2019). Additionally, heteromannans can serve as carbohydrate storage compounds (Meier and Reid 1982; Scheller and Ulvskov 2010). In galls, changes in cell expansion axes and elongation patterns appear to be associated with the spatial distribution of hemicelluloses in the cell walls, which are also related to functional adaptations for nutrient acquisition by the inducing organism, as reported in various gall morphotypes of *Inga ingoides* (Fabaceae) (Bragança et al. 2020).

In recent years, immunocytochemical studies of cell walls in galls have been conducted to identify patterns related to plant developmental responses (e.g., Formiga et al. 2013; Teixeira et al. 2017; Magalhães et al. 2014; Santos et al. 2024; Souza et al. 2024; Ferreira et al. 2025). In this study, we investigated cell wall composition via an immunocytochemical approach in a hemipteran-induced gall model: *Sapium glandulosum* (Euphorbiaceae) galls induced by *Neolithus fasciatus* (Hemiptera: Triozidae). *S. glandulosum* galls are globoid in shape and exhibit histological and cytological compartmentalization within the cortex, along with a high concentration of carbohydrates, which serve as an energy source for sustaining gall development (Rosa et al. 2024). We expect natural redifferentiation followed by maturation of cell walls, as observed in other galls, with a notable increase in structural reinforcement in cell walls (e.g., increasing xyloglucan staining) during gall development, particularly in vascular tissues, since they are the inducer’s feeding site. Changes from typical cell wall development may reflect strategies to evade host defenses, such as inhibiting extensin synthesis.

## Materials and methods

### Host plant–insect system and sample preparation

**Fig. 1:**
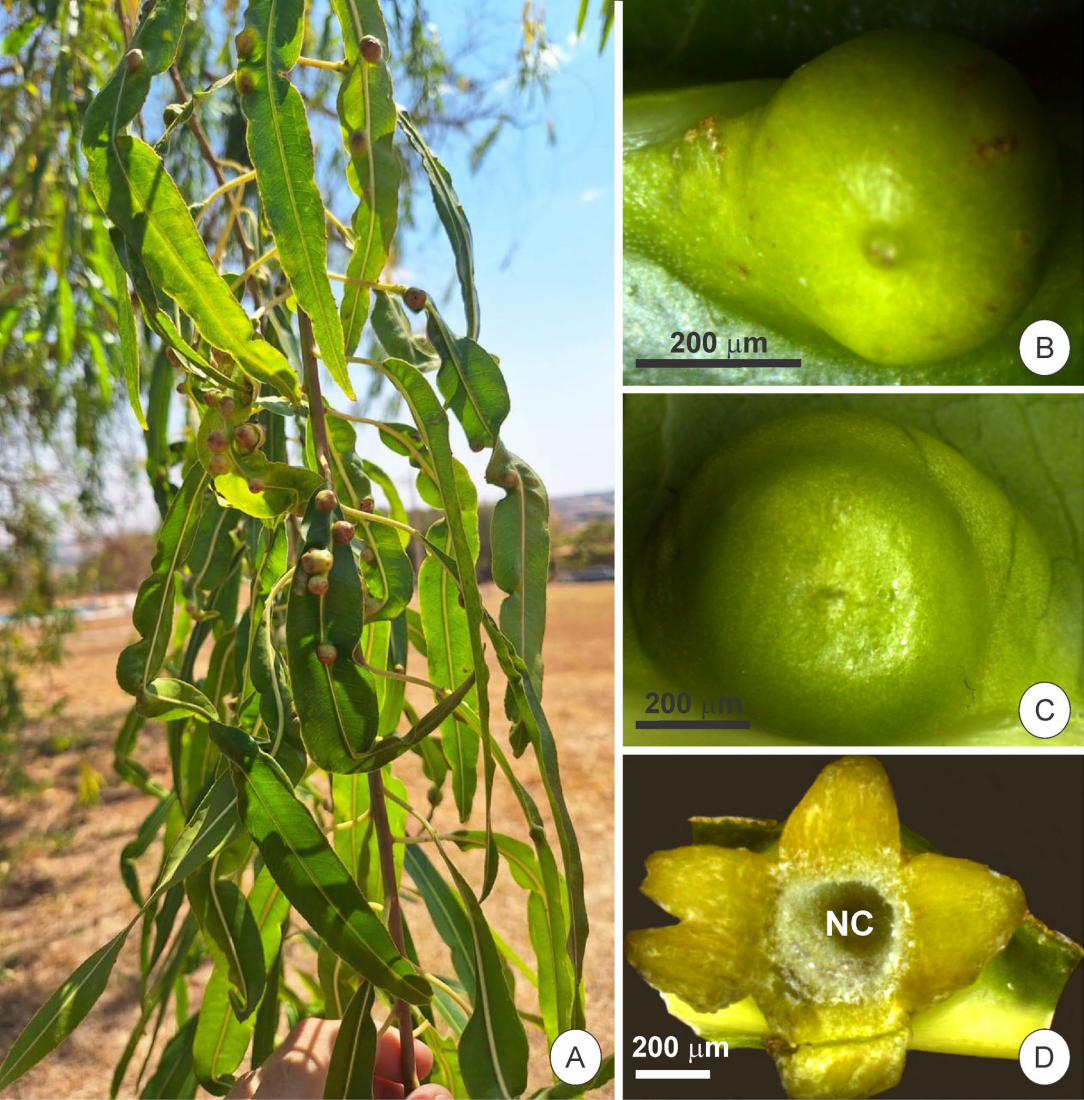
Structural features of *Sapium glandulosum* galls. A- Branch with galls; B- Young gall; C- Mature gall; D- Open senescent gall with nymphal chamber (NC) pointed out

Nongalled leaves and galls were collected from three individuals of *Sapium glandulosum* in the Jataí municipality, Goiás State (17°54’03.6’’ S, 51°45’14.0’’ W), Brazil, within a Cerrado vegetation area. Nongalled leaves were sampled from the third node of the median portion, from the interveinal region. Galls were collected at three distinct developmental stages: young, mature, and senescent. The classification of gall stages was based primarily on external morphology, such as shape and size, and the developmental stage of the inducing insect, *Neolithus fasciatus* (Hemiptera: Triozidae). *Sapium glandulosum* galls develop mainly on the first nodes of the branch, between the first and tenth nodes (Fig. 1a). Young galls (Fig. 1b) were characterized by their small size and the presence of second- to third-instar nymphs. Mature galls (Fig. 1c) had ceased growing and contained nymphs in the fifth-instar stage. Senescent galls (Fig. 1d) were identified by the confirmed absence of the inducer within the nymphal chamber, indicating the end of gall activity. For more information on the development stages of *S. glandulosum* galls, see Rosa et al. (2024).

After collection, fragments of nongalled leaves and galls at different developmental stages were fixed in FAA solution (formalin, acetic acid, and 50% ethanol at a 1:1:18 v/v/v ratio) following Johansen (1940). After 48 h of fixation, the samples were transferred to and stored in 70% ethanol.

### Anatomical analyses

Fragments of nongalled leaves (*n* = 3) and galls (*n* = 3 per developmental stage) were dehydrated through an ethanol series and embedded in 2-hydroxyethyl methacrylate (Historesin^®^, Leica Instruments, Heidelberg). Five-micron-thick transverse sections were obtained via a rotary microtome (Leica^®^ RM2235) and stained with 0.05% toluidine blue at pH 4.7 (O’Brien et al. 1964). The slides were mounted with Entellan^®^ (Kraus and Arduin 1997) and photographed under a light microscope (Leica^®^ DM750) equipped with a digital camera (Leica^®^ ICC50 HD).

### Immunocytochemical analyses

The histological sections obtained from the Historesin^®^-embedded samples were used without staining or permanent mounting. For hemicellulose labeling, the sections were pretreated with a solution containing 10 µg mL⁻¹ pectate lyase (Sigma‒Aldrich) in 2 mM CaCl₂ and 50 mM 3-(cyclohexylamino)−1-propanesulfonic acid (CAPS) buffer (Sigma‒Aldrich, USA), pH 10, for 2 h. Following pretreatment, all the samples underwent the same procedure: the sections were immersed in a blocking solution of Molico^®^ powdered milk in phosphate-buffered saline (PBS) for 30 min. The samples were then incubated for 2 h with primary monoclonal antibodies against LM1, LM2, JIM5, JIM7, LM5, LM6, LM11, LM15, and LM21 (Centre for Plant Sciences, University of Leeds, UK) (Table 1). After being washed in PBS, the sections were incubated in the dark with a fluorescein isothiocyanate (FITC)-conjugated secondary antibody (diluted 1:100 in 3% milk/PBS) for 2 h. After a final PBS wash, the sections were mounted in 50% glycerin. Fluorescence analysis was performed via a Leica^®^ DM4000 LED fluorescence microscope with an HD monochromatic camera (DFC3000 G) and Leica^®^ analysis software. A section from the leaf and each gall developmental stage was processed without primary antibody incubation as a control for nonspecific binding and autofluorescence. In the control, the intensity of the excitation lamp was adjusted until the autofluorescence emission and nonspecific binding were completely suppressed. This adjustment was subsequently used as a reference for the analysis of slides treated with monoclonal antibodies. Also, a FITC filter and a DAPI filter, which mark autofluorescence, were used with excitation wavelengths of 450–490 nm and an emission filter of 515 nm. After that, the overlay function in the microscope software was selected, which generated positive results in green and negative results in blue. Quantitative fluorescence measurements were obtained via ImageJ^®^ software (version 1.51k; http://rsb.info.nih.gov/ij*)*, with the fluorescence intensity evaluated via a grayscale (Gy) analysis method. For this, we used images without the overlapping function, that is, only with positive green markings and negative ones without any sign of fluorescence. For each tissue sample, fluorescence measurements were taken of the cell wall in five cells from each image (*n* = 3 per leaf and gall developmental stage), selecting those that best represented the overall intensity pattern. The average of these measurements was used as the final value for the sample. The fluorescence intensity in the cell walls was categorized as (i) weak (< 15 Gy value), (ii) moderate (15–30 Gy value), or (iii) intense (> 30 Gy value) based on the range of observed values.

**Table 1:** Monoclonal antibodies, epitopes and respective references

Monoclonal antibodies	Epitopes	References
	Proteins	
LM1	Extensins	Smallwood et al. (1996)
LM2	Arabinogalactans	Smallwood et al. (1996); Yates et al. (1996)
Pectins		
LM5	(1 → 4) β-D-galactans	Jones et al. (1997); Andersen et al. 2016)
LM6	(1 → 5) α-L-arabinans	Williats et al. (1998); Willats et al. 2001); Verhertbruggen et al. (2009)
JIM5	Homogalacturanans (HGs) partially methylesterified up to 40% (termed as low methylesterified HGs)	Clausen et al. (2004)
JIM7	Methylesterified HGs – 15% to 80% (termed as methylesterified HGs)	Clausen et al. (2004)
Hemicelluloses		
LM11	Heteroxylans	McCartney et al. (2005)
LM15	XXXG motif of xyloglucans	Marcus et al. (2008)
LM21	Heteromannans	Marcus et al. (2010)

## Results

### Anatomical profile

**Fig. 2:**
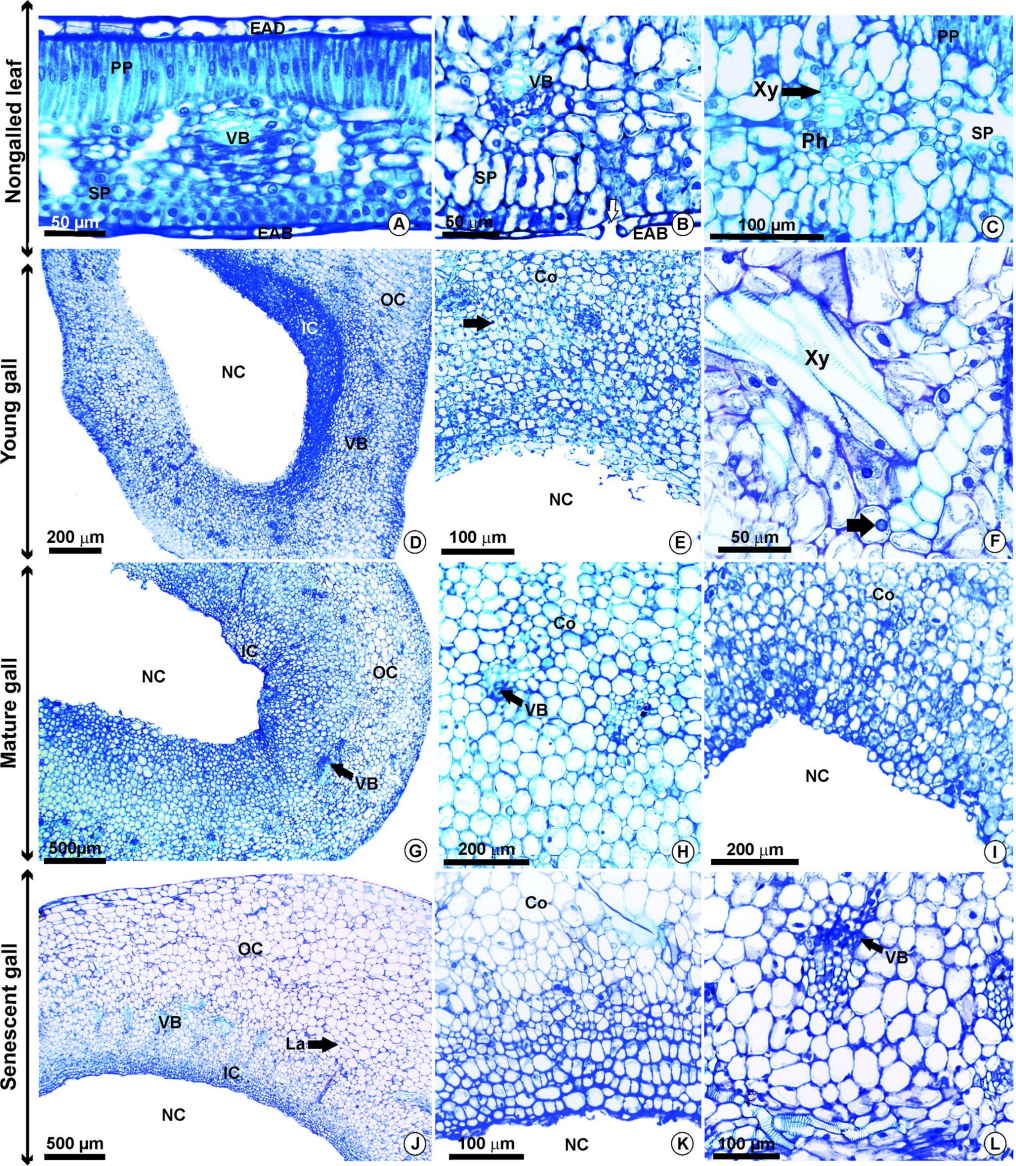
Anatomical features of the nongalled leaves and galls of *Sapium glandulosum*. A-C- Nongalled leaf, with dorsiventral mesophyll, collateral bundles, and stomata (arrow) on the abaxial surface; D-F- Young gall, with parenchymal cortex in formation and a single nymphal chamber. Large and conspicuous nuclei are marked with arrows; G-I- Mature gall, with parenchymal cortex formed, as well as collateral vascular bundles; J-L- Senescent gall, with similarity of tissue organization to the mature gall. *Abbreviations*: EAB- Epidermis on the abaxial surface; EAD- Epidermis on the adaxial surface; Co-Cortex; IC- Inner cortex; La-Laticifer; NC- Nymphal chamber; Ph- Phloem; PP- Palisade parenchyma; OC- Outer cortex; SP- Spongy parenchyma; VB- Vascular bundle; Xy- Xylem

The mature nongalled leaf has a uniseriate epidermis characterized by a thin cuticle and rectangular cells (Fig. 2a). The leaf is glabrous, hypostomatic, and displays dorsiventral mesophyll organization (Fig. 2a). The mesophyll consists of a single, slightly elongated layer of palisade parenchyma and 4 to 5 layers of spongy parenchyma with few intercellular spaces (Fig. 2a, b). The vascular bundles are collateral and well differentiated, accompanied by intrusive growth laticifers (Fig. 2b, c).

The young gall exhibits a uniseriate epidermis with anticlinally elongated cells undergoing hyperplasia (Fig. 2d) and stomata in formation. The cortex is parenchymatic and organized into two distinct regions: (i) an outer cortex composed of elongated cells containing chloroplasts concentrated in the peripheral layers and (ii) an inner cortex made up of smaller, densely packed cells with a cytoplasm-rich profile (Fig. 2d, e). Large and conspicuous nuclei are present especially in cortical cells (Fig. 2e, f). Vascular bundles begin to differentiate in the median region of the cortex (Fig. 2d-f). The nymphal chamber was visible as a developing central cavity (Fig. 2d-f).

The mature gall features a uniseriate epidermis with flattened, tabular cells that are more periclinally elongated than those in young galls (Fig. 2g). The cortex remains parenchymatous and is divided into two regions (Fig. 2g). Nuclei were less evident in all the tissues (Fig. 2h, i). The collateral vascular bundles are centrally located (Fig. 2h), with associated laticifers. The nymphal chamber was still one nymphal chamber (Fig. 2i).

In the senescent stage of the gall, the epidermis shows no major structural differences compared with the mature stage (Fig. 2j). The cortex and vascular tissues retain their previous organization, but the cytoplasmic content in most cortical cells becomes inconspicuous (Fig. 2k, l). At this stage, both cell division and hypertrophy ceased completely (Fig. 2j-l).

### Immunocytochemistry

**Fig. 3:**
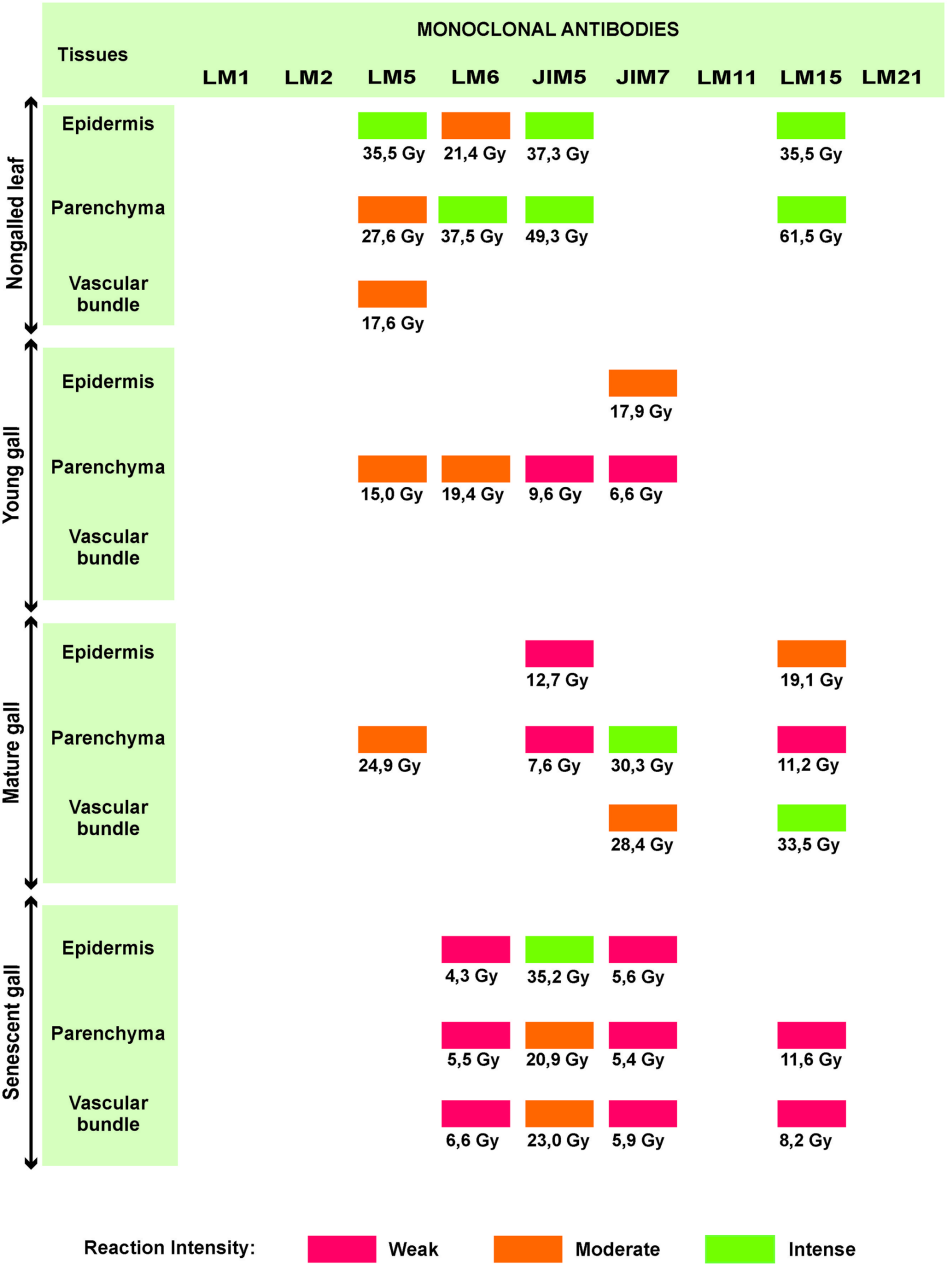
Intensity of immunofluorescence reactions, with grayscale values (Gy), in tissues of nongalled leaves and galls in young, mature and senescent stages of *Sapium glandulosum*

**Fig. 4:**
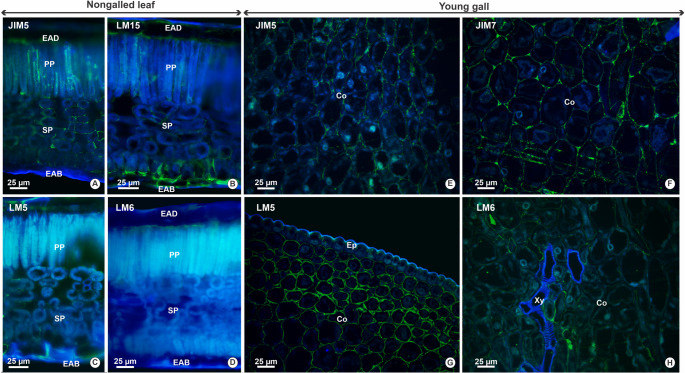
Distribution of pectic and hemicellulosic epitopes in nongalled leaves (**A**-**D**) and young galls (**E**-**H**) of *Sapium glandulosum*. A, E- Low methylesterification HGs, identified by JIM5; B- Xyloglucan epitopes, recognized by LM15; C, G- (1→ 4) β-D-galactan epitopes, labeled by LM5; D, H- (1 → 5) α-L-arabinan epitopes, labeled by LM6; F- Methylesterified HGs, marked by JIM7. *Abbreviations*: Co-Cortex; Ep- Epidermis; EAB- Epidermis on the abaxial surface; EAD- Epidermis on the adaxial surface; PP- Palisade parenchyma; SP- Spongy parenchyma; Xy- Xylem. * Green labelings on the cell wall indicate a positive result, while blue indicates negative results

In nongalled leaves, low-methylesterified homogalacturonan (HG) epitopes, recognized by JIM5, were detected intensely in both epidermal and mesophyll cells (Figs. 3 and 4A). Xyloglucan epitopes, recognized by LM15, showed intense labeling in the epidermis and mesophyll cell walls (Figs. 3 and 4b). The (1→4)-β-D-galactan epitopes recognized by LM5 were moderately to intensely labeled across all the leaf tissues (Figs. 3 and 4c). In contrast, the (1→5)-α-L-arabinan epitopes, which are recognized by LM6, exhibited moderate and intense labeling to epidermal and parenchymal cell walls, respectively (Figs. 3 and 4d).

In young galls, epitopes recognized by JIM5 were weakly detected in the cortical parenchyma cell walls (Figs. 3 and 4e), while JIM7 moderately detected the epitopes in the epidermis (Fig. 3) and weakly in the cortex (Figs. 3 and 4f). Epitopes of (1→4)-β-D-galactan and (1→5)-α-L-arabinan, recognized by LM5 and LM6, respectively, were present only in the cortical parenchyma cell walls, with moderate intensity in both cases (Figs. 3 and 4g and h).

**Fig. 5:**
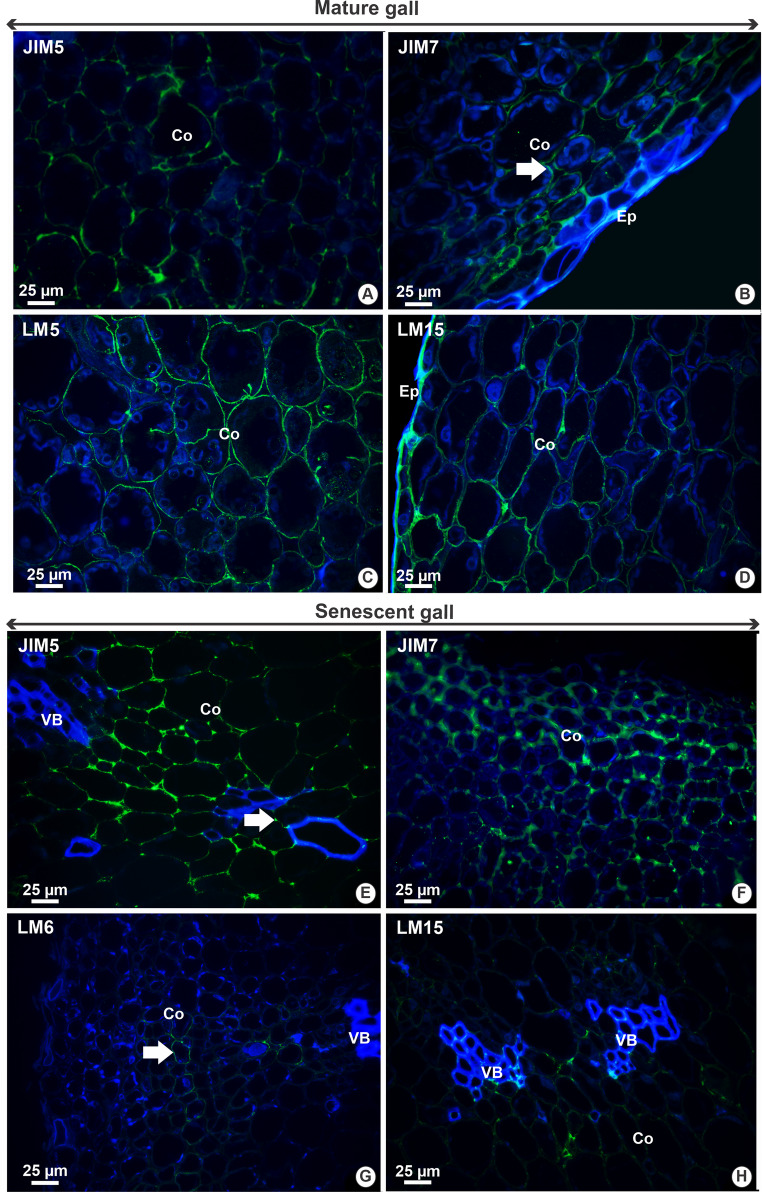
Distribution of pectic and hemicellulosic epitopes in mature (**A**-**D**) and senescent (**E**-**H**) galls of *Sapium glandulosum*. A, E- Low methylesterification HGs identified by JIM5; B, F- Methylesterified HGs labeled by JIM7. Arrow indicates labeling in cortex cells in (**B**); C- (1→ 4) β-D-galactan epitopes recognized by LM5; D, H- Xyloglucan epitopes recognized by LM15. Arrow indicates labeling in vascular bundle cells in (**E**); G- (1 → 5) α-L-arabinan epitopes labeled by LM6. Arrow indicates labeling in cortex cells. *Abbreviations*: Co- Cortex; Ep- Epidermis; VB- Vascular bundle. * Green labelings on the cell wall indicate a positive result, while blue indicates negative results

In mature galls, epitopes recognized by JIM5 were detected weakly in the epidermis (Fig. 3) and cortical parenchyma cell walls (Figs. 3 and 5a). In contrast, JIM7 strongly marked the epitopes in the parenchyma cell walls (Figs. 3 and 5b) and moderately in the vascular bundles (Fig. 3). The epitopes recognized by LM5 showed moderate labeling in the parenchyma cell walls (Figs. 3 and 5c), whereas the epitopes recognized by LM6 were not detected at this developmental stage (Fig. 3). Epitopes recognized by LM15 were detected throughout all the gall tissues, with moderate labeling in the epidermis, weak labeling in the parenchyma (Figs. 3 and 5d), and intense labeling in the vascular bundles (Fig. 3).

The senescent galls exhibited widespread labeling for both pectic and hemicellulosic epitopes, generally with weak intensity across tissues (Fig. 3). The epitopes recognized by JIM5 were intensely labeled in the epidermis cell walls and moderately labeled in the cortical parenchyma and vascular bundles (Figs. 3 and 5e), whereas the epitopes recognized by JIM7 were weakly labeled throughout all the tissues (Fig. 3), especially in cortical cells (Fig. 5f). The epitopes detected by LM6 were weakly labeled in the parenchyma (Figs. 3 and 5g), epidermis, and vascular bundle cell walls (Fig. 3). Epitopes of xyloglucan, recognized by LM15, also display weak labeling in the cortex and vascular bundle cell walls at this developmental stage (Figs. 3 and 5h).

Extensin and arabinogalactan protein (AGP) epitopes, which are recognized by LM1 and LM2, respectively, were not labeled in either nongalled leaf tissues or galls. Similarly, heteromannan epitopes recognized by LM21 were also not detected in any of the analyzed tissues (Fig. 3).

## Discussion

The development of *Sapium glandulosum* galls induced by *Neolithus fasciatus* (Hemiptera: Triozidae) involves the redifferentiation of host leaf tissues, resulting in the formation of a parenchymatic gall, a pattern commonly observed in galls induced by Hemiptera (Ferreira et al. 2019; Rosa et al. 2024). Although these galls are classified as nonnutritive, they exhibit cytological features resembling the nutritive tissues typically found in galls induced by other insect taxa, such as galls induced by Cecidomyiidae in *Manihot esculenta* (Euphorbiaceae) (Souza et al. 2024). In this context, the response of host tissue to biotic stress and subsequent gall development is driven by modifications in cell wall composition, ultimately resulting in the formation of globoid galls composed of cells with novel functionalities. These changes were primarily associated with variations in the degree of pectin methylesterification, which is influenced by the activity of pectin methylesterases (PMEs). PMEs play a crucial role in plant immune responses during biotic interactions by modulating cell wall degradation, thereby contributing to induced resistance in plant tissues (Riseh et al. 2024). Additionally, structural modifications in the side chains of rhamnogalacturonan I (RG-I) and the cross-linking of pectic polymers further impact cell wall properties and defense mechanisms.

The preferred sites for gall induction on *S. glandulosum* are young leaves located at the first nodes. These young leaves exhibit conspicuous nuclei across all tissues, suggesting the persistence of meristematic characteristics (Meier et al. 2017). This observation aligns with the notion that gall-inducing organisms typically target developmentally responsive regions in host plants (Oliveira et al. 2016). In terms of cell wall properties, there appears to be a balance between flexibility and rigidity in most leaf cells. Flexibility (Ulvskov et al. 2005) is indicated by moderate to intense labeling of (1→4)-β-D-galactans across all tissues, whereas rigidity is demonstrated by strong labeling of low methyl-esterified homogalacturonans (HGs) in the epidermis and chlorophyll parenchyma (McCartney et al. 2005; Willats et al. 2001). The lack of labeling of methyl-esterified HGs indicates a natural cell maturation process driven by pectin methylesterases (PMEs), which increase cell wall stiffness (Jolie et al. 2010). The epitope labeling of *S. glandulosum* leaves suggests that the cell walls are still undergoing maturation, even in more developed nodes.

Following gall induction in *S. glandulosum*, there is a notable reduction in the labeling of low methyl-esterified HGs by the JIM5 antibody, which becomes weak in the parenchyma and absent in the epidermis cell walls. Concurrently, methyl-esterified HGs begin to appear in the parenchyma and epidermis cell walls, accompanied by the continued moderate labeling of (1→4)-β-D-galactans in parenchyma cell walls. At this stage, the presence of smaller and more compact cells near the nymphal chamber becomes more evident (Rosa et al. 2024). Although the cortex of *S. glandulosum* galls shows a distinct parenchymatous cell size pattern, the immunocytochemical labeling of the cell wall did not reveal major visible differences compared with the surrounding cells. During cell development, the activity of PMEs promotes the demethylesterification of HGs in the cell wall, leading to new functional properties such as increased stiffness and porosity (Jolie et al. 2010). This enzymatic process has been indirectly observed in several gall systems using an immunocytochemical approach, including leaf galls of *Psidium cattleianum* (Carneiro et al. 2014). However, in some gall systems, such as in galls on *Croton floribundus* (Teixeira et al. 2017), the PME activity appears to be inhibited, resulting in persistent labeling of methylesterified HGs throughout different stages of gall development. Interestingly, *S. glandulosum* does not conform to either of these patterns. In this gall, intense to moderate JIM7 labeling, indicative of highly methylesterified HGs, was observed in the parenchyma and vascular bundle cell walls at the mature stage, suggesting an atypical synthesis of methylesterified HGs in differentiated cells. This pattern of HG labeling indicates the retention of cell wall plasticity and totipotency, which may enable the parenchyma and vascular bundle to have the capacity for future structural modifications. Such parenchyma plasticity has also been documented in eight gall morphotypes of *Croton floribundus* (Teixeira et al. 2022), where parenchyma underwent the most significant structural changes compared with nongalled leaf tissues. *M. esculenta* galls, which are methylesterified HGs recognized by LM20, are intensely labeled in nutritive cells, which is also related to their capacity for cell expansion (Souza et al. 2024). HGs also play a crucial role in the initial defense response of plants to biotic stress by promoting the activity of cell wall-degrading enzymes (CWDEs), which facilitate the breakdown of the cell wall. Among these, PMEs are key enzymes that remove methyl ester groups from pectins, thereby increasing the accessibility of other CWDEs (Pagorelko et al. 2013; Wojtasik et al. 2011; Wolf 2022). The degradation of pectins leads to the formation of oligogalacturonides (OGs), which serve as elicitors of various immune responses in the host plant (Saberi Riseh et al. 2024). In this context, the reduced presence of low methyl-esterified HGs in both young and mature galls suggests the suppression of PME activity. This phenomenon is likely to reflect a strategy by which the gall-inducing organism inhibits host defense signaling, thereby facilitating gall development. Consistently, Takeda et al. (2019) reported up-regulated genes in galls associated with biotic and abiotic stress responses, which contribute to suppressing the plant’s resistance system during gall initiation and development. Notably, this includes the up-regulation of genes related to the pectin methylesterase inhibitor superfamily proteins. Interestingly, PME activity, that is, the demethylesterification process, appears to be reactivated in senescent galls, reinforcing the notion that the galling-inducer actively suppresses PME function during gall developmental stages. In *Matayba guianensis* (Sapindaceae) leaf galls, the demethylesterified HGs were also related to increased vulnerability of galls to the action of pathogens (Silva et al. 2021), as reported in previous studies (Tans-Kersten et al. 1998; Wydra and Beri 2006). This discussion offers a novel perspective for investigating cell wall dynamics and defense modulation during gall formation.

The maintenance of epitopes associated with cell wall flexibility, together with the emergence of methyl-esterified HGs, indicates increased responsiveness of the cell walls in *S. glandulosum* young galls, especially with increasing cell wall elongation capacity (see Albersheim et al. 2010). These results are consistent with the anatomical features observed in young galls, where small cells can be seen as a consequence of hyperplasia and are undergoing cell expansion. However, our immunocytochemical approach contrasts the cytological features of *S. glandulosum* young galls, which exhibit an advanced stage of cell differentiation, with large vacuoles, peripheral cytoplasm, and fully developed chloroplasts (Rosa et al. 2024). Therefore, although the protoplast characteristics of young galls in *S. glandulosum* indicate the onset of cellular differentiation, the cell wall remains responsive to growth.

The maturation of *S. glandulosum* galls was accompanied by structural reinforcement of the cell walls and a reduced capacity for cell elongation, as evidenced by the labeling of xyloglucans with the LM15 antibody. Xyloglucans are the most abundant hemicelluloses in the primary cell walls of eudicots (Scheller and Ulvskov 2010), forming strong associations with cellulose that contribute to the structural integrity of the cell walls (Somerville et al. 2004; Albersheim et al. 2010). This polysaccharide is associated with reduced sliding of cellulose microfibrils, thereby limiting cell wall expansion (Voiniciuc et al. 2018). In galls, xyloglucans are associated with the nutrition of galling herbivores, especially galling inducers that feed on nutritive tissues, as shown for galls of *Inga ingoides* (Fabaceae) (Bragança et al. 2020), *Manihot esculenta* (Euphorbiaceae) (Souza et al. 2024) and *Macairea radula* (Melastomataceae) (Santos et al. 2024). Consistent with Rosa et al. (2024), we found that *S. glandulosum* galls contain abundant neoformed vascular bundles, which serve to increase the number of feeding sites available to the sap-sucking gall inducer (Burckhardt 2005; Carneiro and Isaias 2015), as well as to increase the supply of water and nutrients to the gall tissues. Furthermore, the intense labeling of xyloglucans in the vascular bundles of *S. glandulosum* mature galls appears to reinforce their cell walls, providing structural support to withstand the feeding activity of the galling inducer. Additionally, sap-sucking insects are known to secrete pectinases and hemicellulases to facilitate penetration into plant tissues (Cherqui and Tjallingii 2000; Calderón-Cortés et al. 2012), thereby releasing pentoses, hexoses, and other sugars (Aro et al. 2005). In the context of Hemiptera-induced galls, the continuous feeding activity of the insect may lead to the progressive degradation of cell walls, resulting in the release of sugars into the phloem. These sugars may then become available to insects during feeding, as is viable in the case of gall-inducing *N. fasciatus*.

During the development of *S. glandulosum* galls, rhamnogalacturonan I (RG-I), which contains (1→4) β-D-galactan side chains, was particularly prominent in parenchyma cell walls. LM5 labeled this epitope moderately in young and mature galls. RG-I constitutes approximately 20–35% of the pectic matrix in the cell wall and can perform diverse functions, especially depending on the composition of its side chains (Pérez et al. 2003). The presence of (1→4) β-D-galactan epitopes in cell walls, detected by LM5, has previously been associated with tissue flexibility in plants (Ulvskov et al. 2005) and structural reconfiguration during gall formation, such as in the “kidney-shaped” galls of *Baccharis dracunculifolia* (Oliveira et al. 2014). In *S. glandulosum* galls, sustained LM5 labeling suggests that the cell walls retain expansion capacity, similar to what is observed in nongalled leaves. This finding reinforces the idea of high tissue plasticity within the gall structure (Ferreira et al. 2019). In senescent galls, however, the (1→4) β-D-galactan epitopes are gradually replaced by (1→5) α-L-arabinan epitopes, as indicated by LM6 labeling. This shift likely confers a reinforcement in the cell wall and an increase in cell-to-cell adhesion (Brummell et al. 2004). This distinction in the cell wall composition of senescent galls contrasts with the imperceptible structural differences observed between cell walls of mature and senescent galls, which remain barely apparent at the histological level. Similar LM6 labeling has been reported in the globoid galls of *Croton floribundus* (Teixeira et al. 2017) and *Psidium myrtoides* (Carneiro et al. 2014).

## Main conclusions

The formation and development of galls on *Sapium glandulosum* leaves followed a series of key steps. Initially, the presence of (1→4)-β-D-galactan epitopes in young gall tissues likely contributes to cell elongation, thereby promoting early gall growth. As the galls mature, the labeling of xyloglucans in the vascular bundles suggests a role in reinforcing cell walls, providing the structural support necessary for the feeding activity of the gall-inducing organism. Additionally, the consistently reduced levels of low methyl-esterified homogalacturonans revealed in both young and mature galls indicate suppressed pectin methylesterase activity, a potential strategy to inhibit host defense signaling and thereby facilitate gall development. These findings, in particular, are supported by transcriptomic studies, which have shown the up-regulation of genes related to pectin methylesterase inhibitor superfamily proteins (see Takeda et al. 2019).

## Data Availability

The datasets generated during the current study are available from the corresponding author upon request.
